# HybGFS: a hybrid method for genome-fingerprint scanning

**DOI:** 10.1186/1471-2105-7-479

**Published:** 2006-10-29

**Authors:** Kosaku Shinoda, Nozomu Yachie, Takeshi Masuda, Naoyuki Sugiyama, Masahiro Sugimoto, Tomoyoshi Soga, Masaru Tomita

**Affiliations:** 1 Human Metabolome Technologies, Inc., Tsuruoka, Yamagata, 997-0052, Japan; 2 Institute for Advanced Biosciences, Keio University, Tsuruoka, Yamagata, 997-0017, Japan; 3 Department of Bioinformatics, Mitsubishi Space Software Co. Ltd., Amagasaki, Hyogo, 661–001, Japan

## Abstract

**Background:**

Protein identification based on mass spectrometry (MS) has previously been performed using peptide mass fingerprinting (PMF) or tandem MS (MS/MS) database searching. However, these methods cannot identify proteins that are not already listed in existing databases. Moreover, the alternative approach of *de novo *sequencing requires costly equipment and the interpretation of complex MS/MS spectra. Thus, there is a need for novel high-throughput protein-identification methods that are independent of existing predefined protein databases.

**Results:**

Here, we present a hybrid method for genome-fingerprint scanning, known as HybGFS. This technique combines genome sequence-based peptide MS/MS ion searching with liquid-chromatography elution-time (LC-ET) prediction, to improve the reliability of identification. The hybrid method allows the simultaneous identification and mapping of proteins without *a priori *information about their coding sequences. The current study used standard LC-MS/MS data to query an *in silico*-generated six-reading-frame translation and the enzymatic digest of an entire genome. Used in conjunction with precursor/product ion-mass searching, the LC-ETs increased confidence in the peptide-identification process and reduced the number of false-positive matches. The power of this method was demonstrated using recombinant proteins from the *Escherichia coli *K12 strain.

**Conclusion:**

The novel hybrid method described in this study will be useful for the large-scale experimental confirmation of genome coding sequences, without the need for transcriptome-level expression analysis or costly MS database searching.

## Background

Mass spectrometry (MS) is one of the major proteomics tools of the post-genomic era. Protein identification has traditionally been conducted by peptide mass fingerprinting (PMF) [1-3] or tandem MS (MS/MS) database searching and, while the former performs well with highly purified samples, the latter is the *de facto *standard for identifying proteins in complex samples. However, these approaches are only capable of detecting proteins already listed in databases. PMF implicitly assumes that all genes are annotated and that their complete protein sequences, including post-translational modifications, are known; however, in reality, most of these sequences are inadequately represented in existing databases. As alternative approaches, *de novo *peptide sequencing can be performed by the Edman degradation method [4], MS/MS [5,6], and controlled protein hydrolysis [7]. However, although amino-acid sequences can provide specific information that might be of use in pinpointing gene locations [8,9], *de novo *sequencing generally requires the interpretation of complex MS/MS spectra. The most common method for protein identification is based on the interrogation of MS/MS-based databases. Using this approach, the MS/MS spectra of protein-derived peptide mixtures are matched to theoretical spectra from protein-sequence databases. Search engines, such as Mascot [10] or SEQUEST [11], generate a score reflecting the probability that a given experimental spectrum originates from a particular peptide. The presence of a related protein in the sample of interest is then inferred from these data. However, there remains a need for novel high-throughput approaches, because *de novo *sequencing is not suitable for proteome-wide protein identification, and MS/MS database searching alone is prone to generate both false-positive and false-negative results.

Recently, novel protein-identification methods that are independent of predefined protein databases have been reported [12,13]. In genome fingerprint scanning (GFS), the peptide data are mapped directly onto raw genomic sequences by scanning the PMF data against theoretical peptide masses generated computationally from entire genomes [12]. This approach can identify the genomic loci and amino-acid sequences of proteins. However, the major drawback of this method is that many false-positive matches can be generated, because precursor ion masses alone are used to search the genome-wide peptide-fingerprint data.

To reduce the number of false-positive results, and to improve the efficiency of protein identification, we developed a hybrid method we term HybGFS. This approach uses an extended version of the GFS algorithm that includes the product ion masses and liquid-chromatography elution times as additional parameters for peptide identification. Experimental tandem mass spectra are searched against theoretical peptide data generated from a complete genome sequence, evaluated statistically, and used for genome scanning. In the current study we validated our HybGFS method using a set of 47 recombinant proteins from the *E. coli *K12 strain. A total of 45 (95.7%) of the recombinant proteins were accurately identified and mapped to their correct genomic locations. Based on their amino-acid composition, the peptide LC-ETs were predicted with an artificial neural network (ANN) model and utilized for peptide identification. The HybGFS approach is independent of existing protein databases, in which the coding sequences of proteins are assumed to be established, and simultaneously identifies the genetic loci. Moreover, this novel program does not require the manual interpretation of mass spectra; rather, it uses automatically generated Mascot [10] generic files, thereby allowing high-throughput analysis to be realized. We suggest that this improved method of peptide identification will find many applications in LC-MS-based proteomics.

## Results and discussion

### Generation of an *in silico *peptide database

**Figure 1:**
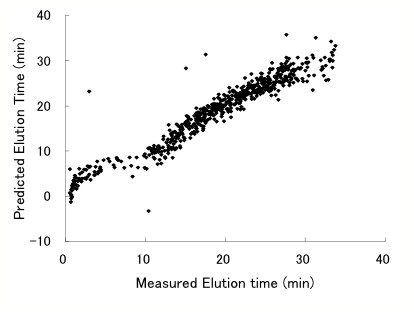
Correlation between experimentally measured and predicted LC-ETs. Data for all peptides in the training dataset obtained through cross validation are shown. The correlation coefficient was 0.9755 and the mean prediction error was 1.52 min (SD = 1.63 min).

We chose *E. coli *K12 to validate HybGFS because extensive genome annotation data are available for this strain and its protein-coding sequences have been accurately determined. To identify proteins without reference to existing databases, we constructed a comprehensive dataset based on genome-sequence information for a total of 2,536,810 *in silico *peptides. We first recorded the amino-acid sequences, the genomic loci and the calculated mass-to-charge ratio (m/z) values of peptides in the dataset. We then included the LC-ETs predicted by the ANN model (see Methods for details). The prediction-accuracy of the trained ANN model is shown in Figure 1. The correlation coefficient for both the predicted and experimental ETs was 0.9755 with a mean prediction error of 1.52 min (standard deviation [SD] = 1.63 min).

### Experimental validation of HybGFS

We tested the accuracy of our method by evaluating its ability to positively identify 47 known protein samples. The recombinant proteins were purified and digested and the resulting peptides analyzed by LS-MS/MS. The resultant raw MS/MS spectra were automatically converted into Mascot generic format (MGF) files using Analyst QS software (Applied Biosystems, CA, USA). We used the precursor/product ion m/z values and the LC-ETs of the peptides in the MGF files to obtain the computational parameters. The m/z error-tolerance values were ± 1 Da for the precursor ion and ± 3.1 min for the LC-ET; the latter was calculated as the sum of the mean prediction error and the SD of the error. Based on these parameters, HybGFS accurately identified 45 (95.7%) of the 47 *E. coli *proteins tested and their genomic loci without reference to information on protein-coding regions.This result confirms that our method is highly effective for determining both protein identity and gene location.

To demonstrate the value of the product ion m/z values and ETs for peptide searching, we repeated these procedures in the absence of one or both of the additional search parameters (Table1). When the precursor ion m/z value alone was used, only 2 of the 47 proteins were correctly identified and the average ranking of the correct protein among the top hits was 157.83. When we used the LC-ET and the precursor ion m/z value together, the number of correctly identified proteins increased to 14 and the average hit ranking was 35.77. When both the precursor- and product ion m/z values were used in the absence of LC-ET information, 38 of the 47 proteins were correctly identified and the average hit ranking was 4.69. When all 3 parameters were applied simultaneously, the results were significantly better: 45 (95.7%) of the proteins were correctly identified and the average hit ranking was 1.85. It should be noted that under the latter condition, the average hit ranking was mainly due to 2 outlier proteins, Eda and PfkA. We further compared our validation results with Mascot search results using the same theoretical ORF database and LC-MS/MS data and identical search parameters, *i.e. *2 missed cleavages, peptide charges (z) of 1–5, error tolerance of ± 1 for precursor, ± 0.8 for product ion, fixed modification of carboxymethyl, and the ESI-QTOF instrument (for detail see Methods). Using Mascot, we found that 41 proteins were correctly identified with top rank and 3 were ranked within the top 4 candidates. The remaining 3 were not identified; 2 of these (Eda and PfkA) were also not identified with HybGFS and considered outliers (Table 1). Mascot search parameters have been debated and we were unable to compare directly and discuss the identification ability of Mascot and HybGFS based only on these results. However, our HybGFS using all 3 parameters (precursor, product ion m/z, and LC-ET) yielded results that were comparable with Mascot algorithms.

**Table 1:** Target protein ranking among the search hit results using precursor ion m/z values, product ion m/z values, and/or LC-ETs.

Gene Product	Rank				
	
	Precursor	Precursor and ET	Precursor and Product	All	Mascot
Average	157.83	35.77	4.69	1.85	-

*PolA*	1	1	1	1	1
*AdhE*	1	1	1	1	1
*PykF*	2	1	1	1	1
*AceE*	7	1	1	1	1
*FumA*	9	1	1	1	1
*AcnA*	9	1	1	1	1
*Pgk*	10	1	1	1	1
*Eno*	12	1	1	1	1
*GapA*	16	1	1	1	1
*Ppc*	21	1	1	1	1
*SucC*	25	1	1	1	1
*Pgi*	30	1	1	1	1
*PtsI*	5	2	1	1	1
*RpiA*	10	2	1	1	1
*SucB*	6	3	1	1	1
*PykA*	8	3	1	1	1
*FumB*	10	3	1	1	1
*IcdA*	11	3	1	1	1
*Gnd*	28	3	1	1	1
*AceA*	301	3	1	1	1
*LpdA*	28	4	1	1	1
*Fba*	282	6	1	1	1
*Zwf*	55	7	1	1	1
*FumC*	211	7	1	1	1
*SucD*	324	7	1	1	1
*TalA*	155	8	1	1	1
*SfcA*	56	9	1	1	1
*SucA*	34	11	1	1	1
*Fbp*	512	11	1	1	1
*GpmA*	34	12	1	1	1
*Acs*	111	14	1	1	1
*PflA*	139	14	1	1	1
*LdhA*	207	27	1	1	1
*PckA*	325	31	1	1	1
*TalB*	493	32	1	1	N.I.
*AckA*	617	50	1	1	1
*Edd*	139	80	1	1	4
*GpmB*	1752	767	1	1	3
*PoxB*	19	2	2	1	1
*Mdh*	62	4	2	1	1
*PflB*	4	1	4	1	1
*PpsA*	27	8	4	1	2
*SdhA*	294	271	7	1	1
*GltA*	129	1	16	1	1
*AceB*	151	13	16	1	1
*Eda*	312	15	23	7	N.I.
*PfkA*	429	15	77	32	N.I.

For comparison, the results searched using Mascot software are also shown. Each recombinant protein's name we experimentally tested in the validation dataset is listed, as is its ranking in the search results.

**Figure 2:**
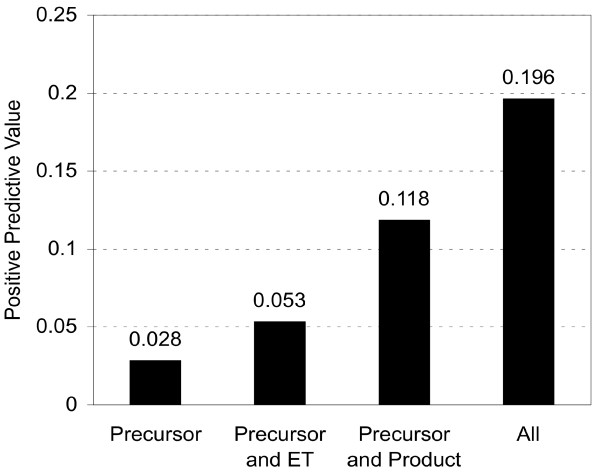
PPV of the peptide-screening algorithm using precursor ion m/z values, product ion m/z values and/or LC-ETs. The PPV corresponds to the ratio of correctly identified peptides (*i.e. *peptide sequences included in the validation protein datasets) to all identified peptides.

To evaluate the identification accuracy at the peptide level, we calculated the positive predictive value (PPV) index, *i.e. *number of true-positive peptide identifications/total number of peptide identifications, of the peptides for each method (Figure 2). We found that the inclusion of all 3 parameters (precursor m/z, product m/z, and ET) yielded the highest PPV index. We also calculated the specificity and sensitivity of the peptide identifications for each matching criterion (Table 2). Due to the large proportion of negative entries in the dataset (peptides) and the difficulties we subsequently encountered in our comparison of specificity, the relative values of the false-positive rate (100-specificity) are also shown. Although sensitivity was high for identifications without product ion m/z (precursor ion m/z; precursor ion m/z and ET), the false-positive rate was also high for these 2 matching criteria. On the other hand, although sensitivity was low for identifications using all 3 indices and for identifications using precursor and product ion m/z, the false-positive rate was drastically (>300 – 700×) improved compared with the results obtained without product ion m/z matching. We ascribe the better result in the correctly identified protein ranking (Table 1) to these low false-positive rates.These results indicate the significant effect of product ion matching on identification. The effect of LC-ET-based screening is also significant as it reduced the false-positive rates from 100.0 to 24.522 (precursor ion m/z and ET), and from 0.134 to 0.073 (precursor, product ion m/z and ET).

Several protein-identification approaches that do not rely on existing databases have been developed previously. For example when Giddings	*et al.*[12] who used the GFS technique searched experimental peptide masses obtained from *Saccharomyces cerevisiae *and *E. coli *against theoretical peptide masses, they were able to identify 17 of the 22 samples tested (77.3%). Taking an alternative approach, Arthur and Wilkins [13] theoretically generated virtual proteins from a genome cleaved into equal-sized fragments, performed translation into all 6 open reading frames (ORFs), and compared the results with experimental data from *Mycobacterium tuberculosis*. However, they found that this method was prone to false-positive peptide matches and consequently, ambiguous protein- and gene-identification results. We opted for a method in which we applied product ion m/z values and LC-ETs to realize efficient peptide identification and conclusive protein inference, and we obtained fewer false-positives. Unlike some of the older methods, HybGFS is highly practical as it does not require the manual interpretation of mass spectra, thus throughput is improved. We suggest that in conjunction with two dimensional (2D)-PAGE and current automated protein spot-handling technologies, the hybrid technique introduced here will be useful for both protein- and gene identification. We also think that our method will prove useful for the annotation of genomes using experimental data and that it will assist in the discovery of novel gene products – goals that standard bioinformatics gene-searching algorithms often fail to achieve.

**Table 2:** Specificity and sensitivity of peptide identifications using precursor ion m/z values, product ion m/z values, and/or LC-ETs.

	**Sensitivity**	**Specificity**	**FP Rate (Relative Value)**
**Precursor**	41.208	96.038	100.000
**Precursor + ET**	35.317	99.028	24.522
**Precursor + Product**	8.467	99.995	0.134
**All**	9.789	99.997	0.073

Relative values of the false-positive (FP) rate are also shown. The highest FP rate (precursor) was normalized to 100 and other FP rates were represented as the ratio to the normalized value. We used this representation of the FP rate to facilitate easier comparison.

In addition to 3 indices we utilized, other LC-MS-derived information that reflects the physicochemical properties of peptides may be usable in HybGFS. For example, Parker [14] employed peak intensity information to improve the accuracy of peptide identification in MALDI-MS-based proteomics. Although a method for the prediction of peptide peak intensity for ESI-MS remains to be established, we could use this information for more accurate peptide identification. In addition, the high-resolution mass spectrometric approach is useful. The utility of accurate mass tag- and LC-ET prediction in large-scale peptide identification has been discussed elsewhere [15]. Our HybGFS would further reduce false-positive peptide matches if high-resolution MS were used in conjunction with LC-ET prediction.

### Scalability of HybGFS to other organisms

In higher eukaryotes, *e.g. *human and rat, the size of individual introns is much larger than in other eukaryotes (human: 3413.4 bp; rat: 1091.7 bp; *Schizosaccharomyces pombe*: 92.7 bp, (mean)) [16]. Furthermore, higher eukaryotes have multiple introns (human: 4.0 ± 4.3; rat: 3.0 ± 3.3/gene) [16], whereas a yeast gene usually has at most one intron [17]. Due to difficulties in evaluating peptide localization on the genome, the application of GFS methodology to higher eukaryotes comprised of multiple exons remains a considerable challenge [12]. It is inherently difficult to identify by the current GFS methodology peptides translated over exon/exon junctions and the existence of alternative splicing variants increases the possibility of such false-negatives. At least 74% of human multi-exon genes are alternatively spliced [18] and in mouse protein-coding transcripts, 79% of splice variations altered the protein product [19]. Therefore, from a practical perspective, current GFS methods including our HybGFS are not scalable to higher eukaryotes at present. However, HybGFS is not limited to bacteria; we consider it applicable to lower eukaryotes such as yeast and *Aspergillus*, because the intron size and frequency in these organisms are small [16,17].

## Conclusion

We present a new method for mapping peptide sequences onto their genomic positions using LC-MS/MS data and genome-sequence information. HybGFS does not require predefined protein databases or annotated ORF information. Our validation results indicate that the use of product ion m/z values and peptide LC-ETs in addition to precursor ion m/z values, as employed in the original GFS method, significantly reduces the number of false-positives, a major problem in the traditional GFS method that results in errors in both mapping and protein identification. HybGFS will therefore be useful for large-scale experimental confirmation of coding sequences in genomes without the need for transcriptome-level expression analysis or costly MS database searches.

## Methods

### Pre-compilation of *in silico *peptides

**Figure 3:**
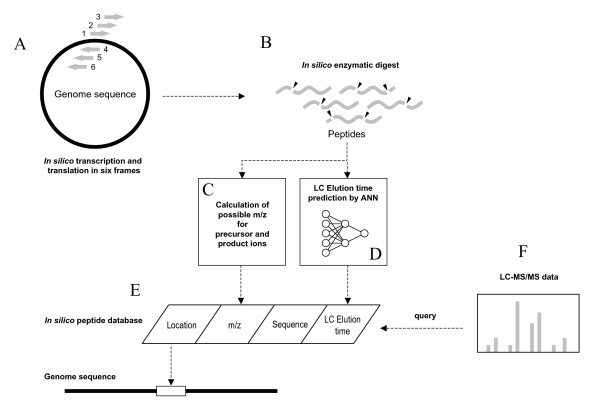
Schematic flowchart depicting the HybGFS method. (**A **and **B**) Generation of *in silico *peptide sequences from a genome sequence. (**C**) Calculation of precursor and product ion m/z values for *in silico *peptides. (**D**) LC-ET prediction for *in silico*-generated peptides using ANNs. (**E**) Pre-compilation of *in silico *peptide information into a database. (**F**) Querying experimental peptide data obtained by MS/MS to the database.

All possible peptide sequences were generated *in silico *using the procedures described below. The genome of the *E. coli *K12 strain (GenBank:NC_000913) was downloaded via the National Center for Biotechnology Information (NCBI) ftp server on January 12, 2004. The sequence was computationally translated and cleaved according to the rules for the LysC endopeptidase (Figs. 3A and 3B). Translation of the genome was conducted in all 6 reading frames, irrespective of the annotated ORFs. The acceptable number of missed cleavage sites was set at ≤2. For each *in silico*-generated peptide, the m/z values of the precursor/product ions, the amino-acid sequences, and the genomic location were stored in our *in silico *peptide database (Figure 3C). To calculate the m/z values of the precursor ions, we assumed that the peptide charge (z) was between 1 and 5. The b-y series ions, which were the most abundant under our experimental conditions, were used to list the m/z values of all possible product ions for each precursor ion. In addition, the LC-ETs were predicted using the ANN model (Figure 3D).

### ANN model

We developed an ANN model, coupled with a genetic algorithm-based normalization, according to the basic method described by Petritis *et al. *[20]. The model consisted of 20 input nodes, 2 hidden nodes, and 1 output node. The 20 input vectors corresponded to the numbers of each of the 20 amino-acid residues making up the peptides. Each input vector was normalized to the maximum value among all peptides in the training sets; these comprised 614 well-known peptides identified by MS/MS under the same LC-gradient conditions. To reduce the output error, the 20 input vectors for these peptides were repeatedly entered into the ANN model using a back-propagation algorithm. The ANN model was trained using 500 (81.4%) of the 614 peptides and its predictive power was evaluated by cross-validation with the remaining data. The correlation coefficient of each validation was calculated using least-square approximation and the ANN weightings with the best correlation coefficients were adopted to predict the LC-ETs of all the *in silico *peptides.

After these procedures the genomic loci, precursor/product ion m/z values, and predicted LC-ETs for all of the peptides were calculated and entered into our *in silico *peptide database (Fig. 3E). All experimental LC-MS/MS data were queried against this database (Fig. 3F).

### Experimental sample preparation and MS

To prepare a validation set for query against HybGFS, His-tagged proteins were produced using the 'A Complete Set of *E. coli *K-12 ORF Archive' (ASKA) library [21]. Clones were grown in LB medium at 37°C. After 3-h incubation, 1 mM isopropylthiogalactoside (IPTG) was added to induce the expression of the recombinant proteins. Cells were harvested by centrifugation at 5,000 rpm for 10 min, suspended in buffer containing 50 mM sodium phosphate and 300 mM NaCl (pH7.0), sonicated on ice, and stored at -80°C. Protein extracts were centrifuged at 8,000 rpm for 20 min at 4°C and the supernatant was applied to a Talon metal-affinity chromatography column (Clontech, CA, USA) to purify the His-tagged proteins. These were eluted in lysis buffer containing 200 mM imidazole. Protein samples (12.5 μg) were reduced with 1 μl reduction buffer (1 μg/μl dithiothreitol in water) and alkylated with 1 μl alkylation buffer (5 μg/μL iodoacetamide in water). After dilution with Milli-Q water, the proteins were enzymatically digested overnight at room temperature with 10 AU/mL LysC endopeptidase. The resulting peptides were then separated on an Agilent 1100 series LC system with a Zorbax SB-C 18 3.5-μm reverse-phase column (2.1 × 50 mm; Agilent Technologies, Palo Alto, CA) packed with 1.8-μm particles. The mobile phases for the gradient elution were water (A) and acetonitrile (B), both with 0.2% formic acid, and the peptides were eluted from the column using 1–45% gradients of solvent (B). The column eluent was transferred to an electrospray-ionization quadrupole/quadrupole time-of-flight (QqTOF) tandem mass spectrometer (QSTAR; Applied Biosystems, CA, USA) operated in the positive-ion mode. MGF files containing peak lists and m/z and LC-ET information for each ion were automatically generated by Mascot.dll script in Analyst QS software (Applied Biosystems).

### Identification of proteins and gene loci

Experimental peptide-fingerprint data for each protein sample were queried against the *in silico *peptide database as follows. *In silico *peptides were initially searched using the m/z values of the precursor ions and the associated ETs. Error tolerances were set at ± 1 Da for the precursor ions and at ± 3.1 min for the LC-ETs. *In silico *peptides within these ranges were selected. Positively-matched peptides were further screened by MS/MS matching based on b-y series ions. For each candidate precursor ion, a list of N product ions with random m/z values was generated, with N corresponding to the number of experimentally observed product ions. The number of random product ions was limited to between 200 and 1,000, which corresponded with the selected MS scan range. The theoretical product ions (b-y series) for each *in silico *peptide entry were compared with both the random- and the experimental sets. Specifically, a confidential limit of number of random peptide matches was calculated from the result of 100-fold random matching. If the number of matches with the set of experimental product ions was greater than the 95% upper confidence limit, the *in silico *peptide was selected. Finally, the selected *in silico *peptides were mapped back to the genome sequence according to their position stored in the database.

### Potential coding-sequence generation

A total of 153,202 possible ORFs (from start- to stop codons including alternative ORFs) found in the *E. coli *genome were generated in all 6 reading frames, irrespective of any previous annotation. Each putative ORF was initially scored using the Z-transformed number of experimental peptides that mapped to it. The putative ORFs were further screened by comparing their estimated MW with the known MW of sample proteins. The MWs (Da) of the putative ORFs were estimated based on the linear correlation with length (L, bp) using the equation MW = 181.4 + 36.9 L. This formula was derived from documented ORF-and protein-sequence information held in the NCBI GenBank database. ORFs with a total calculated MW that differed by more than 4,000 Da from that of the experimental proteins were eliminated. The resulting putative ORFs were assumed to encode the queried sample proteins. The search algorithm was implemented using Perl version 5.8.

## Authors' contributions

KS conceived the method, carried out the validations, and drafted the manuscript. NY and MS helped to design the validation. TM and NS carried out the protein preparation and mass spectrometry. TS and MT supervised the work. All of the authors read and approved the final manuscript.
